# Applications of single-cell RNA sequencing in rheumatoid arthritis

**DOI:** 10.3389/fimmu.2024.1491318

**Published:** 2024-11-12

**Authors:** Marxa L. Figueiredo

**Affiliations:** Department of Basic Medical Sciences, College of Veterinary Medicine, Purdue University, West Lafayette, IN, United States

**Keywords:** single-cell, RNA-sequencing, rheumatoid arthritis, scRNA-seq, RA

## Abstract

Single cell RNA sequencing (scRNA-seq) is a relatively new technology that provides an unprecedented, detailed view of cellular heterogeneity and function by delineating the transcriptomic difference among individual cells. This will allow for mapping of cell-type-specific signaling during physiological and pathological processes, to build highly specific models of cellular signaling networks between the many discrete clusters that are present. This technology therefore provides a powerful approach to dissecting the cellular and molecular mechanisms that contribute to autoimmune diseases, including rheumatoid arthritis (RA). scRNA-seq can offer valuable insights into RA unique cellular states and transitions, potentially enabling development of novel drug targets. However, some challenges that still limit its mainstream utilization and include higher costs, a lower sensitivity for low-abundance transcripts, and a relatively complex data analysis workflow relative to bulk or traditional RNA-seq. This minireview explores the emerging application of scRNA-seq in RA research, highlighting its role in producing important insights that can help pave the way for innovative and more effective therapeutic strategies.

## 1 Introduction

### 1.1 Overview of rheumatoid arthritis and therapeutic targeting challenges

Rheumatoid arthritis (RA) is a chronic autoimmune inflammatory disease affecting 0.5-1% of the global population annually (1). It is characterized by the destruction of synovial tissue, resulting in thickening of the synovium and infiltration of immune cells and synovial fibroblasts. The chronic inflammation and extra-articular manifestations lead to pain, joint damage, and disability (2). Recent research using single-cell RNA sequencing has uncovered several new subclusters of cells, cytokines, and chemokines that are involved in RA, enhancing our understanding of its pathology and progression.

The diagnosis and treatment of RA are challenging, despite the use of markers such as anti-citrullinated peptide antibodies and rheumatoid factors to differentiate between seropositive and seronegative RA. Most recent reports have introduced new markers including anti-carbamylated protein and anti-lysine acetylated antibodies. The challenge remains in how to integrate these discoveries into routine clinical practice. For example, mass cytometry has added CD4, CD45RA, and CD11c as potential markers for RA diagnosis, along with the functional markers p-p38 and CD86, yet the application of these findings in treating diverse patient populations is still evolving (3). And despite recent advances in targeted therapies, such as TNF inhibitors and IL-6 blockers, there remain significant concerns regarding their long-term efficacy and safety, including potential adverse effects that include an increased infection risk (4, 5). And while these treatments have transformed RA management for many patients, they do not achieve remission for everyone, and a significant proportion of patients still experience incomplete or variable responses.

Genetic studies have suggested that sequence variations impact seropositive RA more significantly, which complicates personalized treatment approaches. Of significant promise, multiomics approaches have identified potential therapeutic targets including CCL2 and MMP13, but translating these into effective treatments and accurate diagnostics presents ongoing challenges (6). The integration of new inflammatory phenotypes and predictive markers into clinical practice remains a significant hurdle for improving RA management. It has been traditionally difficult to predict the cell types and signaling/crosstalk in the affected joints in order to target and treat RA because of the heterogeneous and dynamic nature of the infiltrate and the multiple levels of interaction possible in the joint. Among the most pressing questions in the field of RA (and other arthritis research) is which immune cells drive disease versus which promote resolution. Rigorous characterization of the immune cell dynamics and the relative contribution of specific cellular subsets to disease processes including cartilage damage and bone erosion in RA will facilitate the development of targeted, disease-modifying therapies. Also, accurately defining the temporal dynamics, ontogenies, and functions of distinct immune cell subtypes in the synovium will be critical if these cells are to be targeted for treatment. Also to be considered is the trajectory of immune infiltrates once in the RA joint, now possible through single-cell RNA sequencing approaches.

### 1.2 Emergence of scRNA-seq

Single-cell RNA sequencing (scRNA-seq) is a cutting-edge technology that provides a detailed view of cellular heterogeneity and function by investigating transcriptomic variation at the individual cell level. This approach enables the identification of distinct cell types during physiological and pathological processes, constructs detailed cellular signaling models, and reveals regulatory networks among the identified cell clusters. These capabilities offer many advantages relative to bulk RNA sequencing (RNA-seq), which averages gene expression across a cell population and might miss rare or transient cell types. ScRNA-seq provides detailed insights into cellular states and transitions, although it also presents challenges such as higher costs, lower sensitivity for detecting low-abundance transcripts, and complex data analysis relative to bulk RNA-seq. We explore here how scRNA-seq can advance our understanding and treatment of RA, potentially leading to new insights and therapeutic breakthroughs.

In most RA studies, researchers have typically sequenced tens of thousands to hundreds of thousands of individual cells to achieve comprehensive coverage of cellular heterogeneity. Moreover, the sequencing depth often ranges from 10,000-100,000 reads per cell, depending on the goals of each study and the complexity of the tissue being analyzed. This level of sequencing depth ensures the detection of rare cell populations and subtle expression differences, which are crucial for understanding the diverse roles of various cell types in RA pathology (7). By capturing a broad spectrum of gene expression profiles, these studies have provided detailed insights into the cellular mechanisms driving RA, enabling more precise identification of therapeutic targets and the development of tailored treatment strategies. This technology is highly relevant to RA research, as it provides insights into a condition marked by high cell heterogeneity, which is often masked in bulk tissue analyses of synovium. This immune cell heterogeneity will be discussed next in more detail.

## 2 Immune cell heterogeneity in RA

Prior research has shown that although targeted therapies, such as TNF inhibitors and IL-6 blockers, have transformed RA treatment (2), many patients do not achieve remission (8), highlighting the need for new therapeutic targets. Variable responses to targeted therapies indicate that RA is a highly heterogeneous condition (9). Interestingly, genetic and clinical differences in disease duration or activity traditionally have not enabled reliable predictions of the treatment response or the key druggable targets (2, 10). Therefore, a more detailed understanding of the cell states and phenotypes in the synovial joint is needed to better inform prognosis and therapeutic targets for RA. In fact, clinical trials using bulk-RNA-seq or immunohistochemical target detection reveal that treatment response indeed may depend on the particular cellular composition at the inflamed joint (11, 12). Moreover, previous studies have identified specific immune cell states that are promising as therapeutic targets, such as HBEGF^+^IL1B^+^ macrophages, PD-1^hi^ T peripheral helper cells (T_PH_), and NOTCH3^+^ synovial fibroblasts (13–21), all of which contribute to RA pathophysiology. These findings offer new avenues for personalized therapy in RA that may also offer insights into additional conditions featuring joint inflammation, such as other arthritis types.

### 2.1 Macrophages

scRNA-seq has revealed significant functional diversity among macrophages in the RA synovium. For instance, CX3CR1^+^ macrophages have been identified that form protective barriers to isolate the joint and limit inflammation (22). In contrast, RA treatments that often target inflammatory macrophages, also influence fibroblast behavior, and may perpetuate inflammation (14). Recent work by Aliverini et al. demonstrated that targeting specific macrophage clusters or activating transcription factors that drive remission could enhance therapeutic efficacy (17). These findings indicate promise for more personalized and effective treatment strategies by focusing on the functional roles of different macrophage subpopulations. Another recent report by Alivernini et al. profiled synovial tissue macrophages (STM) that persist in remission, and examined how they might contribute to joint homeostasis in patients with early RA, treatment-refractory RA, relative to RA in remission (17). Four distinct subpopulations of STMs were identified, of which two had unique remission-specific transcriptomic signatures enriched in negative regulators of inflammation, MerTK^+^TREM2^hi^ and MerTK^+^LYVE1^+^. These STM populations were enriched in lipid mediators that could help resolve inflammation and promote repair responses by synovial fibroblasts in culture. Interestingly, while these STM populations were associated with inflammation resolution, their presence in patients in remission paradoxically correlated with an increased risk of disease flare after therapy cessation. This suggests that targeting or modulating these specific subpopulations may offer novel therapeutic strategies to manage RA and potentially prevent relapse, yet more research is needed to fine-tune the desired responses.

### 2.2 Fibroblasts

The application of scRNA-seq in several recent studies has significantly enhanced our understanding of fibroblast phenotype and function in RA joints. Two key fibroblast subclusters have been recently described, CD55^+^, which protect the synovium from immune complex-mediated arthritis, and CD90^+^, which aid in extracellular matrix organization (23). Mizoguchi et al. expanded this understanding by describing a subset of PDPN^+^CD34^–^THY1^+^ fibroblasts surrounding blood vessels in RA patients, that are believed to facilitate immune cell infiltration and sustain chronic inflammation (24). Chronically activated fibroblasts are known to degrade excessive matrix and destroy cartilage, leading to permanent joint damage by activating osteoclasts (25). They promote inflammation and cartilage destruction by recruiting T cells and producing cytokines such as IL-6, central to RA pathogenesis. These fibroblasts also contribute to the formation of ectopic germinal centers and exacerbate inflammation (26, 27). Additionally, fibroblasts recruit and support B cell survival through the secretion of various factors (28, 29), and also influence macrophage activity, worsening joint inflammation and bone destruction (30, 31).

Zhang et al. reported novel fibroblast subclusters with distinct functions, including THY1^+^CD34^−^HLA^-^DR^hi^ fibroblasts that express high levels of IL-6, and DKK3^+^ fibroblasts that help prevent cartilage degradation (13). Croft et al. characterized FAPα^+^ fibroblasts, revealing that FAPα^+^THY1^+^ fibroblasts modulate inflammation, whereas FAPα^+^THY1^-^ fibroblasts contribute to bone and cartilage damage (32). Recent work also suggests that targeting NOTCH3 in CD90+ fibroblasts may reduce inflammation and joint damage (19). Moreover, the development of specific small molecule agonists that activate melanocortin type 1 receptor has been reported to induce the regression of arthritis by influencing fibroblast activation (33). Recent findings by Stephenson et al. identified three subpopulations of RA synovial fibroblasts: CD55^+^ and CD90^+^ subsets, among others. CD55^+^ fibroblasts, located in the intimal lining, are involved in synovial fluid formation and turnover. They express high levels of hyaluronan synthase 1 (HAS1), lubricin (PRG4), and DNASE1L3, which contribute to joint homeostasis and anti-inflammatory responses (13, 23, 34, 35). HAS1, in particular, is upregulated by TGF-β and is important for cartilage lubrication. CD55^+^ fibroblasts also contribute to endothelial cell proliferation and modulation of reactive oxygen species responses (23). Taken together, these findings indicate there is a high level of molecular complexity in RA fibroblast subsets, providing a strong rationale for the development of targeted therapies that can modulate fibroblast function to treat and possibly prevent joint destruction in RA.

### 2.3 Chondrocytes and bone cells

Chondrocytes and bone cells, which are essential for cartilage maintenance and bone remodeling, are significantly affected in RA. In the RA joint, chondrocytes often exhibit abnormal behavior, contributing to cartilage degradation and joint damage. scRNA-seq studies have identified distinct chondrocyte subpopulations with altered gene expression profiles, for example upregulation of matrix metalloproteinases and other catabolic enzymes including MMP-1, 2, 3, 7, 9, 13, and 14, leading to the breakdown of extracellular matrix components (36). Inflammatory cytokines such as IL-1β and TNF-α worsen these processes by promoting chondrocyte apoptosis and suppressing matrix synthesis. Understanding the functional states of chondrocytes in RA can inform therapeutic strategies aimed at preserving cartilage integrity and preventing disease-associated joint damage.

Bone cells, including osteoblasts, osteoclasts, and osteocytes, play a critical role in the bone destruction observed in RA. Osteoclasts are highly active in RA, leading to increased bone resorption and joint erosion. scRNA-seq has revealed distinct osteoclast subpopulations with varying levels of activity and gene expression related to bone resorption, such as RANKL and NF-kB signaling pathways. Osteoblasts and osteocytes may have impaired function in RA, contributing to insufficient bone formation and repair. Osteoblasts in RA often show altered expression of key bone-forming proteins, while osteocytes can release signals that further activate osteoclasts and influence bone remodeling (37, 38). Recent studies have highlighted specific fibroblast subpopulations in RA synovial tissue that affect bone cells. CD34^–^THY1^–^ fibroblasts, found predominantly in the lining area of the synovium, express BMP-6 (24, 39), which is associated with enhanced osteoblastic bone formation. Additionally, CD34^+^ fibroblasts, located in both superficial lining and deeper sublining areas, contribute to joint inflammation by secreting many inflammatory factors. CD34^–^THY1^+^ fibroblasts create a perivascular zone in the deep sublining layer and are involved in T cell transport by overexpressing TNFSF11, further influencing bone and cartilage pathology. Overall, targeting these bone cell interactions could help balance bone resorption and formation, potentially focusing on modulating osteoclast activity, enhancing osteoblast function, or correcting osteocyte signaling to provide new avenues for maintaining joint integrity.

### 2.4 Lymphocytes and eosinophils

Immune cells such as T cells, NK cells, eosinophils, monocytes, and B cells also play pivotal roles in RA, and their interactions offer potential therapeutic targets. For instance, distinct T cell populations, such as CD8^+^ cytotoxic T cells and PD-1^hi^CXCR5^-^CD4^+^ T cells, found within synovial tissue, contribute to RA severity (13). CD8^+^ T cells are involved in direct cytotoxic effects and tissue damage in the joint environment, while PD-1^hi^CXCR5^-^CD4^+^ T cells, including peripheral helper T (T_PH_) and follicular helper T (T_FH_) cells, interact with B cells in the synovium, influencing their recruitment, activation, and differentiation. These T cells support B cell differentiation and antibody production, potentially exacerbating inflammation. Targeting these T cell interactions could provide a strategy to modulate disease progression and alleviate symptoms.

Regulatory T cells (T_regs_), characterized by markers such as FOXP3, are crucial for maintaining immune tolerance and regulating inflammation both in circulation and within synovial tissue. In RA, the balance between pro-inflammatory T cells and T_regs_ can influence disease severity. T_regs_ are involved in suppressing excessive immune responses and promoting tissue repair in the synovial microenvironment. Understanding their function in the RA microenvironment and exploring their potential as therapeutic targets could offer new approaches for managing disease activity and promoting remission. Natural Killer (NK) cells, involved in inflammatory signaling through interferon (IFN) pathways (40), present another therapeutic target. Dysregulated NK cell activity, whether in circulation or within the synovium, can exacerbate inflammation and joint damage in RA. Potential therapeutic strategies could involve targeting IFN signaling pathways in NK cells to help control inflammation.

Regulatory eosinophil subsets have been identified as beneficial in resolving chronic arthritis and promoting tissue regeneration within the synovial microenvironment (41), indicating that enhancing their function could offer new avenues for therapy. Monocytes and B cells are also crucial; proinflammatory IL1B^+^ monocytes and NR4A-expressing B cells, whether circulating or localized in the synovium, represent potential biomarkers and therapeutic targets for chronic autoantigen stimulation. Plasma cells contribute to the chronicity of RA by producing autoantibodies such as rheumatoid factor and anti-citrullinated protein antibodies. The differential immunological mechanisms between anti-citrullinated peptide antibody-positive and -negative RA further highlight the need for tailored therapeutic strategies. Overall, targeting specific immune cell subsets (both circulating and within the synovium) and their interactions, as well as modulating signaling pathways, holds promise for advancing RA treatment and managing its diverse manifestations more effectively.

### 2.5 Neutrophils

Neutrophils are often among the first responders to inflammation and have been implicated in RA pathology. They can contribute to joint damage through the release of reactive oxygen species (ROS) and proteolytic enzymes, which can lead to tissue destruction and exacerbate inflammation (42). The functionality of neutrophils in RA patients is frequently altered, which can influence disease progression and severity (43). For instance, studies have shown that neutrophils from RA patients exhibit increased production of ROS and enhanced formation of neutrophil extracellular traps (NETs), which are implicated in the inflammatory processes of RA (42). Moreover, profiling granulocytes, including neutrophils, presents challenges due to the presence of high intracellular RNases that complicate gene expression analysis (43). However, recent research has revealed distinct subpopulations of neutrophils with varying functional capacities (43). Notably, low-density neutrophils have been correlated with pathology in multiple inflammatory diseases, including RA, suggesting that these cells may play a significant role in disease mechanisms. In addition to neutrophils, type 2 immune responses, characterized by increased eosinophils, can exert protective effects in murine models of arthritis. This protection is mediated through IL-4 and IL-13 signaling, which activates the STAT6 pathway, leading to modulation of the immune response (44). Profiling granulocyte-associated gene patterns, such as markers of phagocytosis and complement activation, in synovial samples from RA patients could provide further insights into the contribution of these cells to inflammatory arthritis (43). Understanding the specific roles and functional states of neutrophils and other granulocytes in RA may pave the way for novel therapeutic strategies aimed at better modulating the inflammatory environment.

### 2.6 Dendritic cells

Dendritic cells (DCs) are pivotal in the immune response, particularly in the context of RA, where they serve as crucial antigen-presenting cells that bridge the innate and adaptive immune systems. DCs facilitate the activation and differentiation of T cells, which can exacerbate inflammatory processes. The presence of mature DCs in the rheumatoid synovial fluid and tissues highlights their role in sustaining the inflammatory cascade by secreting chemokines that attract T cells and presenting antigens to autoreactive T cells (45, 46). This interaction is essential for the development of the RA autoimmune response. Various subsets of DCs, including conventional DCs (cDCs) and plasmacytoid DCs (pDCs), have been identified in RA. cDCs are instrumental in driving immune responses by efficiently capturing and processing antigens, which they present to T cells, thereby promoting T cell activation and differentiation (45, 47). In contrast, pDCs are known for their ability to produce large quantities of type I interferons, contributing significantly to the chronic inflammation observed in RA (47, 48). Recent research has explored therapeutic approaches aimed at modulating the functions of DCs in RA. Strategies that inhibit the pro-inflammatory actions of cDCs or enhance the regulatory functions of other DC subsets could provide promising avenues for altering disease progression (48, 49). For instance, inducing tolerogenic DCs through innovative methods, such as using modified nanoparticles, has shown potential in regulating immune responses and mitigating inflammation in RA (48). Furthermore, DC-based vaccines and treatments targeting DC-T cell interactions are being investigated as potential strategies to modify the immune response in RA, aiming to restore balance and reduce autoimmunity (45, 49). The distinct functions of DCs in RA highlight the complexity of the immune response, and emerging therapeutic strategies targeting these cells offer hope for more effective management of the disease.

### 2.7 Endothelial cells

Endothelial cells play a critical role by modulating inflammation and vascular changes. In RA, these cells become activated and upregulate adhesion molecules (e.g. VCAM-1 and ICAM-1), which facilitate the recruitment and adhesion of immune cells to the inflamed synovium (50). The upregulation of these adhesion molecules is crucial for the extravasation of inflammatory cells, including T cells, monocytes, and neutrophils, into the joint space, thereby exacerbating the inflammatory response (51). This process is further supported by findings that endothelial activation leads to increased vascular permeability, allowing for a greater influx of inflammatory cells and proteins into the joint (52). In addition to their role in immune cell recruitment, activated endothelial cells contribute to synovial hyperplasia through angiogenesis, which is driven by pro-inflammatory cytokines and growth factors such as VEGF and IL-6 (53). The neovascularization associated with RA not only enhances blood flow but also increases the delivery of inflammatory mediators to the site of inflammation, perpetuating the cycle of inflammation and joint damage (53). Moreover, endothelial cells interact with immune cells by secreting various cytokines and chemokines, which further influences the recruitment and activation of these immune cells. Therapeutic approaches aimed at inhibiting endothelial cell activation, blocking adhesion molecules, or disrupting angiogenesis have shown promise in reducing inflammation and joint damage in RA (53). Recent studies have highlighted the potential of targeting endothelial cell-derived factors and pathways to develop novel treatments for RA. For instance, inhibiting the interactions between endothelial adhesion molecules and immune cells could mitigate the inflammatory environment and slow disease progression (54). Additionally, therapies that target the processes driven by activated endothelial cells may provide a dual benefit by reducing both inflammation and the associated synovial hyperplasia.

## 3 Cell-type abundance phenotypes (CTAPs)

**Figure 1:**
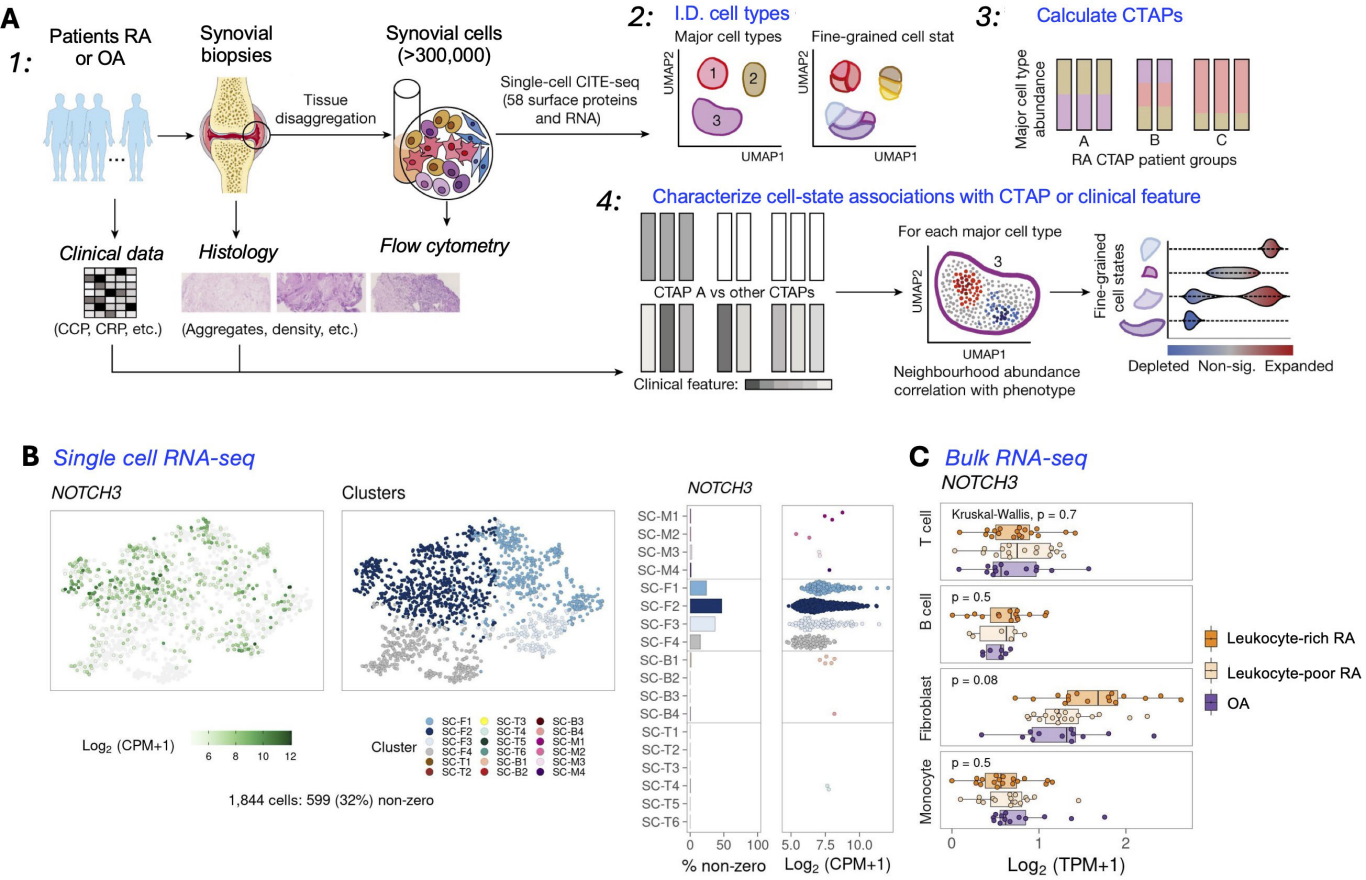
Overview of a multi-modal single-cell synovial tissue pipeline and cell-type abundance analysis revealing distinct RA CTAPs. **(A)** Summary of a study combining clinical and histology metrics, synovial sample processing, and computational analyses to identify fine-grained cell states. **(B)** The RA CTAPs can be queried in detail using a resource available to the public (56). UMAPs can be generated that integrate scRNA-seq or Bulk RNA-seq **(C)**, and also mass cytometry results (not shown) to discriminate major cell types. Cluster annotations : Fibroblasts (CD45- Podoplanin+); SC-F1: CD34+ sublining fibroblasts; SC-F2: HLA+ sublining fibroblasts; SC-F3: DKK3+ sublining fibroblasts; SC-F4: CD55+ lining fibroblasts; Monocytes (CD45+ CD14+); SC-M1: IL1B+ pro-inflammatory monocytes; SC-M2: NUPR1+ monocytes; SC-M3: C1QA+ monocytes; SC-M4: IFN-activated monocytes; T cells (CD45+ CD3+); SC-T1: CCR7+ CD4+ T cells; SC-T2: FOXP3+ Tregs; SC-T3: PD-1+ Tph/Tfh; SC-T4: GZMK+ CD8+ T cells; SC-T5: GNLY+ GZMB+ CTLs; SC-T6: GZMK+/GZMB+ T cells; B cells (CD45+ CD3- CD19+); SC-B1: IGHD+ CD270 naive B cells; SC-B2: IGHG3+ CD27- memory B cells; SC-B3: autoimmune-associated cells (ABC); SC-B4: Plasmablasts. [**(A)** Adapted from ref. 55, licensed under Creative Commons Attribution 4.0 International License. **(B)** Example from utilizing the website: https://immunogenomics.io/ampra/ (56)].

Recent pioneering work by Zhang et al has defined RA tissues in an innovative and comprehensive manner, stratifying tissues into six groups, referred to as cell-type abundance phenotypes or CTAPs (55). Each of the CTAPs was characterized by enriched cell states, demonstrating the diversity of synovial inflammation in RA. These ranged from samples enriched for T and B cells to samples lacking lymphocytes, for example. A summary of this analysis is detailed in **Figure 1A**, showing a study which has identified many distinct cell states across myeloid, stromal, and endothelial cells within the RA synovium. Metrics that could be correlated with certain CTAPs included disease-relevant cell states, cytokine expression, risk genes, and histology and serology markers. This “atlas”, made possible by scRNA-seq technology, indicated that the CTAPs were very dynamic and could predict treatment response, indicating high utility in classifying RA synovial phenotypes. The “atlas” was developed in the initiative called Accelerating Medicines Partnership-Autoimmune and Immune-Mediated Diseases (AMP-AIM), a collaboration among 20 organizations from the public, private, and not-for-profit sectors, managed by the FNIH. Building on the success of the prior initiative, AMP RA/SLE, which used advanced technologies to study disease at the single-cell level in lupus-affected kidneys and arthritic joints, AMP RA Phase I has identified novel cell populations and pathways, potentially offering new therapeutic targets.

**Figure 2:**
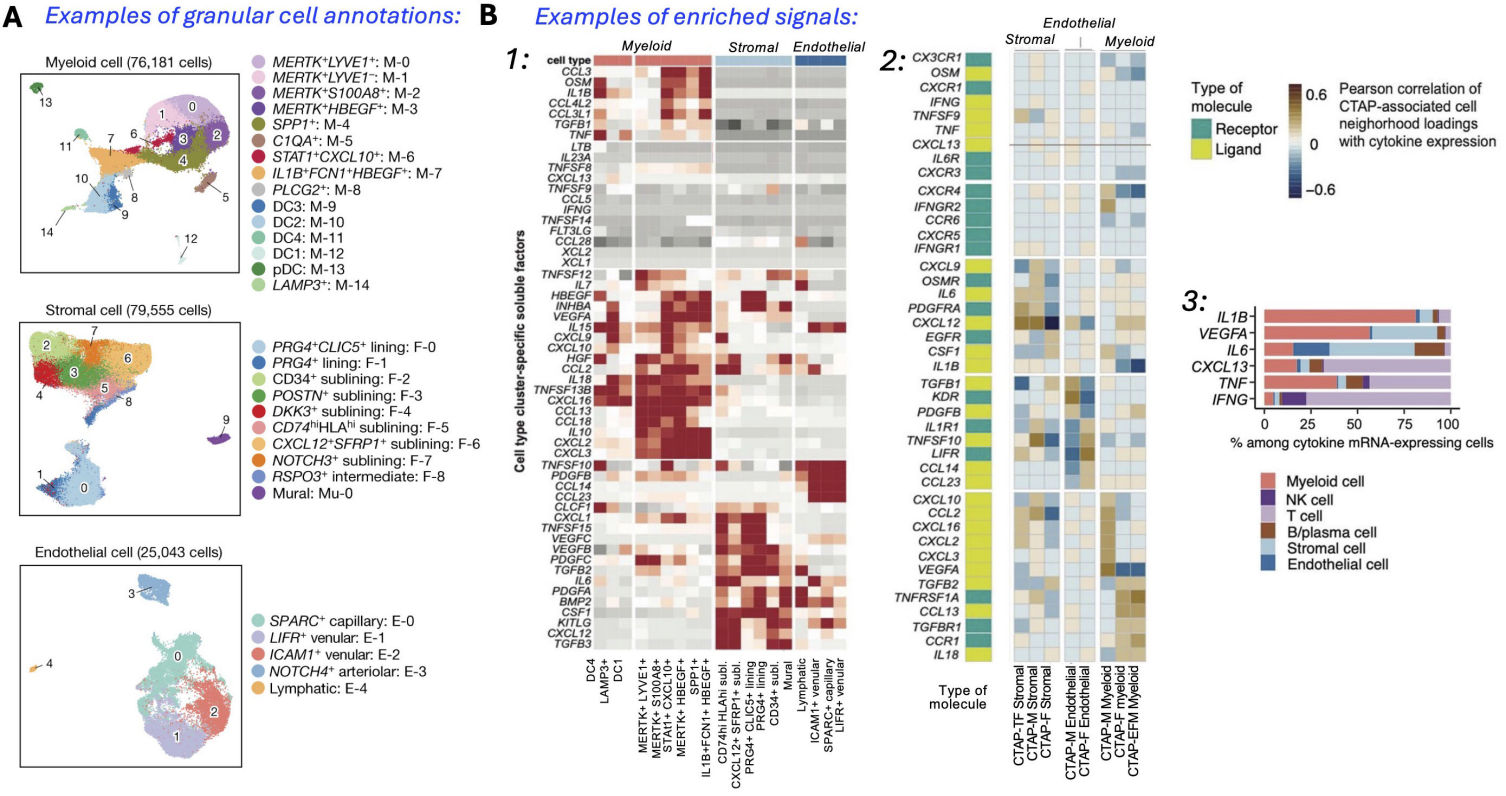
Single-cell analysis identifies a multitude of distinct cell states in rheumatoid arthritis synovium. **(A)** Reference UMAPs for myeloid, stromal, and endothelial cells, colored by fine-grained cell-state clusters. **(B)** Enriched signals (1): Heatmap of soluble factors specific to cell type clusters; (2) Expression UMAPs with cells colored from blue (low) to yellow (high), and aggregate heatmap of cytokines and receptors correlated (r > 0.5) with CTAP-associated cells, hierarchically clustered by CTAPs with receptor/ligand labels; (3) Percent contribution of mRNA-expressing cells from major cell types. [Adapted from ref. 55, licensed under Creative Commons Attribution 4.0 International License].

Interestingly, some results from these studies can be readily accessed (57), and contain insights from scRNA-seq, bulk RNA-seq, and mass cytometry (workflow in **Figures 1B, C**). In the same study, the heatmaps and UMAPs presented correlated visually the enriched soluble factors and cytokine/receptor expressions with specific CTAP-associated cells. Examples of the cell type granularity, and cytokines and signaling pathways possible to identify with these approaches are shown in **Figure 2**. Collectively, the insights from the CTAP data could help inform novel targeted treatments. Furthermore, if these insights can be aligned with current scRNA-seq information derived from mouse models of RA, we anticipate that the connection between preclinical and clinical therapeutic agents could be strengthened for the more efficient translation of preclinical results.

Also interesting would be the relevance of several of the CTAP findings and analysis workflow to other arthritis types. For instance, Liao et al. found that fibroblasts in OA were associated with processes such as protein processing, glycolysis, and proteoglycans in cancer, while RA fibroblasts, were linked to focal adhesion and ECM receptor interactions (58). Additionally, RA patients had significantly fewer B cells but more CD8^+^ T cells and neutrophils relative to OA patients. Cai et al. identified shared regulatory effects of the Wnt, TGFb, FcRI, and ERBB signaling pathways on synovial fibroblasts in both RA and OA (58), suggesting overlapping pathogenic mechanisms and new therapeutic targets for managing disease progression. Finally, several parallels in the RA cell types and signaling were also identified in post-traumatic OA, particularly in macrophage subtypes, in a recent study by Knights et al. (59), suggesting therapeutic target discovery in RA by scRNA-seq could be of high utility for other arthritis types, and vice-versa.

## 4 Future directions

In summary, scRNA-seq has significantly enhanced our understanding of RA by revealing the heterogeneity, marker genes, and signaling pathways of chondrocytes, synovial cells, and immune cells. It provides insights into the lineage and developmental relationships among these cells. Notably, scRNA-seq has identified new cytokines and signaling pathways, such as JAK/STAT, which are crucial for RA pathology and validate potential therapeutic targets. Additionally, the technology has highlighted specific fibroblast and macrophage subpopulations, suggesting that targeting these subsets could be a promising approach for treatment. Despite impressive results, scRNA-seq still holds immense potential for future refinement. Unidentified or low-percentage cell populations, including synovial cells, osteoblasts, endothelial cells, and mesenchymal cells, might be underrepresented in some datasets. Further exploration of cellular subset changes and their phenotypes at various developmental stages is needed. Integrating scRNA-seq with multiomics (proteomics, metabolomics) could help clarify intercellular networks and disease mechanisms, while traditional methods like flow cytometry and mouse (CIA, CAIA, STIA) and rat models (CIA) of RA should validate key findings. Continued development of scRNA-seq platforms may lead to breakthroughs in RA treatment by targeting newly identified cytokines, signaling pathways, and specific cell subpopulations.

Other emerging single-cell analysis technologies are transforming RA research by characterizing cellular and molecular dynamics. Techniques including single-cell ATAC-seq can enhance our understanding of regulatory regions within individual cells, and spatial transcriptomics will help overcome the limitations of traditional single-cell transcriptomics by mapping RNA distribution in tissue sections. Single cell multiomics integrates genomics, transcriptomics, and proteomics at a high resolution, facilitating the identification of cell subtypes, lineage tracing, and detailed genetic analysis. Collectively, these methods, as they become more cost-effective and mainstream, promise to advance our knowledge of mechanisms and improve personalized treatment strategies for RA. Future developments in the field should focus on improving the resolution and sensitivity of scRNA-seq technologies, integrating spatial transcriptomics to better understand tissue-specific dynamics, and addressing the challenges of translating multiomics insights into clinical applications. scRNA-seq integration with multiomics will likely lead to innovative diagnostic tools and personalized therapies, driving more effective and targeted treatment approaches for RA and ultimately improving patient outcomes.

## 5 Conclusion

scRNA-seq has significantly advanced our understanding of RA by revealing novel cell populations, markers, and mechanisms underlying disease processes. This technology provides detailed insights into cellular heterogeneity, disease mechanisms, and cell interactions, offering potential new therapeutic targets. However, its application faces notable challenges, including difficulties in preparing single-cell suspensions from hard tissues including bone and cartilage, high costs limiting sample size, and issues with batch effects and data interpretation. Addressing these limitations through improved experimental designs, integrating scRNA-seq with other technologies (multiomics), and fostering research collaborations will enhance its utility and accelerate advancements in RA research.
